# Effect of Liuzijue on pulmonary rehabilitation in patients with chronic obstructive pulmonary disease: study protocol for a multicenter, non-randomized, prospective study

**DOI:** 10.1186/s12906-022-03789-6

**Published:** 2022-11-17

**Authors:** Jiaming Hu, Rundi Gao, Yiting Wang, Yan Li, Yaqin Wang, Zhen Wang, Junchao Yang

**Affiliations:** 1 Zhejiang Chinese Medical University, Binwen Road 548, Binjiang District, Zhejiang, 310053 Hangzhou China; 2 The Second Clinical Medical College, Zhejiang Chinese Medical University, Binwen Road 548, Binjiang District, Zhejiang, 310053 Hangzhou China; 3 Department of Respiration, The First Affiliated Hospital of Zhejiang Chinese Medical University, Youdian Road 54, Shangcheng District, Zhejiang, 310006 Hangzhou China

**Keywords:** Chronic obstructive pulmonary disease, Liuzijue, Pulmonary rehabilitation

## Abstract

**Background:**

Traditional Chinese exercise as a new pulmonary rehabilitation technique has been increasingly used and achieved good results in pulmonary rehabilitation of chronic obstructive pulmonary disease (COPD). The aim of this study is to investigate the protective effects of Liuzijue on exercise tolerance, lung function, and quality of life in patients with COPD.

**Methods:**

This study is a multicenter, non-randomized, prospective study. Patients will be divided into a control group (CG) and a Liuzijue group (LG) based on their willingness to learn Liuzijue. None of the outcome assessors will know the grouping of patients. Participants in this study will be collected from stable COPD patients who are outpatients or inpatients in 3 centers in China since September 2021. Patients will meet the diagnostic criteria for GOLD stage I-II COPD (FEV_1_% ≥ 0.5 and FEV_1_/FVC < 0.7) and be aged 40 years or older. Patients voluntarily will take part in the clinical study and sign an informed consent form. All participants will follow their existing medication. For LG patients, Liuzijue training has been added. Patients will practice Liuzijue for more than 30 minutes a day, more than 5 days a week, and adhere to the training for 3 months. Outcome indicators are 6-minute walk test (6MWT), lung function (FEV_1_%, FEV_1_/FVC, MMEF, PEF), modified British Medical Research Council (mMRC) score, COPD assessment test score (CAT), acute exacerbations and changes in drug treatment.

**Discussion:**

This study quantified the effect of Liuzijue on the pulmonary rehabilitation of COPD patients in the stable phase of the disease, and provided a basis for the use of Liuzijue in COPD patients.

**Trial registration:**

Chinese clinical trial registry, ChiCTR2100048945. Date: 2021-07-19. http://www.chictr.org.cn/showproj.aspx?proj=129094

**Supplementary Information:**

The online version contains supplementary material available at 10.1186/s12906-022-03789-6.

## Background

Chronic obstructive pulmonary disease (COPD) is a group of respiratory diseases characterized by airflow limitation. It has a progressive course with high morbidity and mortality. However, outcomes of drug treatment remain limited. It therefore causes a huge family and social burden and is an important public health problem [1]. An epidemiological survey of COPD in China published in The Lancet in 2018 found that the prevalence of COPD in people aged 40 years or older was 13.7% [2]. WHO predicts that by 2030, 4.5 million people will die from COPD each year, making it a major global public health problem [3, 4]. The focus of COPD treatment has gradually shifted from the acute exacerbation phase to the stabilization phase. How to further prevent and treat COPD so as to improve the quality of life of patients has been widely concerned by society and the medical community.

The American Thoracic Society (AST) and the European Respiratory Society (ERS) have recommended early pulmonary rehabilitation using exercise training for COPD patients [5]. Pulmonary rehabilitation has many advantages, such as improving life quality and exercise capacity, improving psychological conditions and reducing mortality [6]. However, less than 10% of COPD patients have access to pulmonary rehabilitation services [7, 8]. There are many reasons for this phenomenon, such as shortage of healthcare professionals, single exercise modality, the need for special equipment and fixed places, and the cost of equipment maintenance. All these problems have led to low patient compliance with exercise training, reducing the accessibility and feasibility of pulmonary rehabilitation [9–13]. Therefore, a convenient and effective rehabilitation technique is needed to meet the rehabilitation demand of COPD patients.

Liuzijue originated from Taoism in China. The earliest treatise on the Liuzijue was first published in Tao Hongjing’s “The Yang Xing Yan Ming Lu” during the Northern and Southern Dynasties, about 1500 years ago. In 2007, Liuzijue was reorganized by the General Administration of Sport of China and formed a complete set of standard qigong training methods. This method covers aspects of modern pulmonary rehabilitation, such as exercise training, respiratory muscle training and psychological rehabilitation, and meets the basic requirements of pulmonary rehabilitation. The characteristics of the Liuzijue are: (1) Liuzijue is to exhale through reading the specific 6-word of “xu”, “he”, “hu”, “si”, “chui”, and “xi”. (2) The Liuzijue uses deep and slow breathing and reverse abdominal breathing to exercise the function of the respiratory muscles. (3) The physical movements in Liuzijue exercise the muscles of the whole body, especially the skeletal muscles of the upper and lower limbs. (4) Liuzijue enhances the patient’s mental conditioning during the training process.

Safety, efficiency, and economy are the strengths of Liuzijue. In terms of safety, the movements of Liuzijue are simple and easy to perform, the exercise load is not too heavy and the amount and duration of exercise is easy to control. In terms of effectiveness, the rehabilitation effects of Liuzijue have been confirmed by several studies. Meta-analyses have shown that Liuzijue improves the St. George’s respiratory questionnaire score (SGRQ), COPD assessment test score (CAT), modified British Medical Research Council (mMRC) score, 6-minute walking test (6MWT), percentage of forced expiratory volume in 1 second to predicted value (FEV_1_%), and ratio of forced expiratory volume in 1 second to forced vital capacity (FEV_1_/FVC) in COPD patients [14–16]. The results show that Liuzijue could improve the quality of life, exercise tolerance and lung function in COPD patients. In terms of economy, learning Liuzijue is inexpensive, with a few movements and easy to master. There is no requirement for venue and facilities for Liuzijue, which is easy to implement and low-cost.

Therefore, in this study, Liuzijue is used for pulmonary rehabilitation exercises in stable COPD patients. This study investigates the effects of Liuzijue on exercise endurance, lung function, quality of life in COPD patients. This will lead to the search for a more effective, inexpensive and easy method of respiratory rehabilitation training to be applied to patients in the community and at home.

## Methods/design

### Study design

**Fig. 1:**
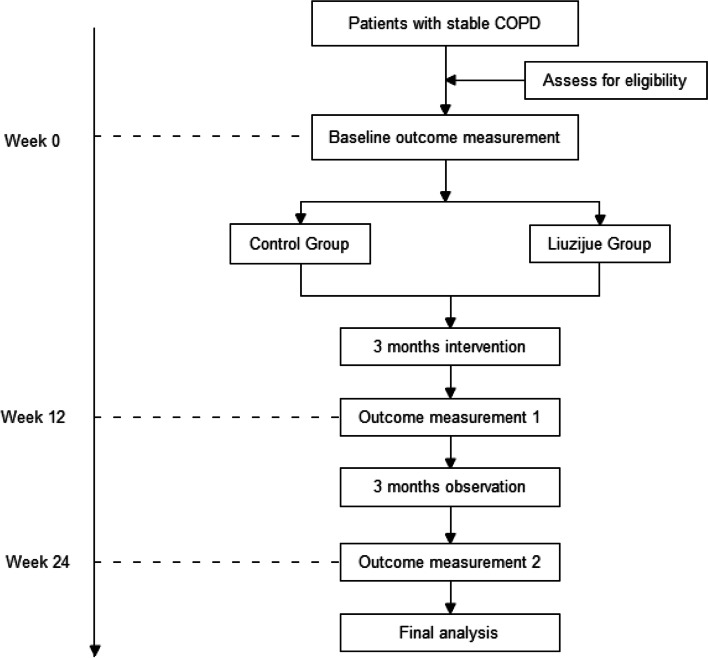
Study flow chart

**Fig. 2:**
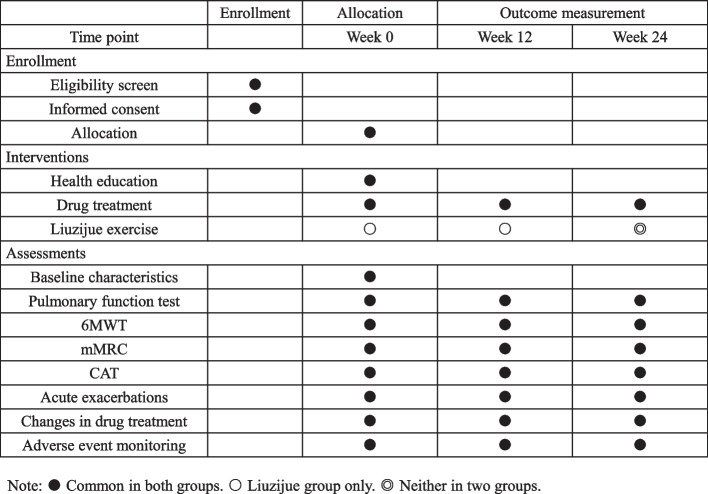
Illustration of the design

This study is a multicenter, non-randomized, prospective study. There are 3 centers in this study, including the First Affiliated Hospital of Zhejiang Chinese Medicine University, the Affiliated Hospital of Jiangxi University of Traditional Chinese Medicine and the Affiliated Hospital of Chengdu University of Traditional Chinese Medicine. Patients will be divided into a control group (CG) and a Liuzijue group (LG) based on their willingness to learn Liuzijue. None of the outcome assessors will know the grouping of the patients. Participants in this study will be collected from stable COPD patients who are outpatients or inpatients since September 2021. The protocol has been registered in the China Clinical Trial Registry (registration number: ChiCTR2100048945) and approved by the Ethics Committee of Zhejiang Provincial Hospital of Chinese Medicine (Ethics number: 2019-KL-095-06). Informed consent will be obtained from all participants before they participate in the study. The research process is shown in Fig. 1, and the time point of the research is shown in Fig. 2. The protocol follows the SPIRIT 2013 checklist, provided in Additional file 1: SPIRIT 2013 checklist.

### Sample size

The sample size is calculated based on the primary outcome (6MWT) using PASS15.0 software. According to the previous literature [17], the mean for the control group is estimated to be 418.71 with a standard deviation of 66.19. After Liuzijue exercising, the mean for the experimental group is 483.62 with a standard error of 67.30. With a power of 80% and an alpha of 5% (two-sided), the minimum sample size per group is 23. Given a drop-out rate of 20%, the minimum sample size in total is 46.

### Participants

Patients diagnosed with stable COPD in the outpatient or inpatient department of 3 centers from September 2021 are enrolled in this study. Individuals interested in the study participate with the study.

Inclusion criteria: (1) Meet COPD diagnostic criteria. (2) FEV1% ≥ 0.5, and FEV1/FVC < 0.7 (3) Age above 40 years. (4) Patients voluntarily participated and signed an informed consent form. Exclusion criteria: (1) Patients with serious primary diseases such as cardiac, hepatic, renal, cerebral and hematopoietic system. (2) Patients with localized giant bullae of lung, pneumothorax and extreme physical weakness or disability. (3) Pregnant or lactating women. (4) Psychiatric patients. (5) Unwilling to accept study measures or otherwise unable to cooperate. (6) Patients who are currently participating in other drug-related clinical trials or intervention studies.

### Intervention

All patients continue to take their current medication and receive health education on the self-management of COPD. Patients are advised to quit smoking and get vaccinated against pneumonia and flu. LG group receive 60 minutes of Liuzijue course, where patients learn Liuzijue through video and face-to-face instruction. Patients then exercise Liuzijue at home for 30 minutes or more a day, more than 5 days a week for 3 months.

### Follow-up

General information and observations will be collected from patients at the time they join the study. For the LG group, follow-up visits will be made once a week (telephone or WeChat) for the first 1 month and once every 2 weeks (telephone or WeChat) for the next 2 months after enrolment. By means of follow-up visit, patients will be reminded to exercise Liuzijue, and researchers can get to know the changes in their health condition and promptly help them to overcome the difficulties encountered in the process of exercising. Field follow-up visits is conducted 3 months and 6 months after admission to record changes in the patient’s outcome indicators for both CG and LG.

Patients will be followed up using a WeChat applet. The applet will help patients to clock in for training, do self-test health scales, upload and view test reports, initiate consultations with doctors, view health information and record acute exacerbations. Through the platform, doctors can view patients’ records such as punch cards, scale assessments and test reports, and contact patients for follow-up visits. At the end of each training session, patients will be asked to make a record on the applet. Furthermore, the number of follow-up visits is flexibly adjusted according to the patient’s punch card records.

### Outcome measurement

The primary outcomes is 6-minute walking test (6MWT). Secondary outcomes include pulmonary function tests (FEV1%, FEV1/FVC, MMEF, PEF), CAT score and mMRC score. Pulmonary function tests are performed by specialists. 6MWT is performed by trained research team members. Symptoms of breathlessness will be assessed using the mMRC and the CAT questionnaire will be used to assess quality of life. If the patient has an acute exacerbation, it will be recorded. Any changes in drug treatment of the patients will also be recorded. None of the outcome assessors will be aware of the patients’ grouping.

### Statistical analysis

Statistical analysis of the data will be performed using SPSS Statistics 26.0. Continuous variables with normal distribution will be described by mean and standard deviation. Continuous variables with non-normal distribution or ordinal variables will be described by median and interquartile range. Categorized variables will be summarized as counts and proportions. Continuous variables will be compared using Student’s t-test (normal distribution) or the Mann-Whitney U test / Wilcoxon rank test (abnormal distribution). Ordinal variables will be compared using Mann-Whitney U test/ Wilcoxon rank test. The categorical variables will be compared using Fisher’s exact test or the chi-squared test. Training-related effects will be assessed using a repeated ANOVA. All reported *P* values are two-sided, and *P* values < 0.05 is considered statistically significant.

### Safety

Patients will be asked to stop training and contact the researchers immediately when they find any discomfort. Researchers will evaluate the situation and give corresponding treatment.

### Data management

Medical data collected from participants will be stored on the data platform (https://copd.daoqihz.com/), which is especially made for this study.

## Discuss

What is particularly striking about Liuzijue is its focus on breathing. Its unique breathing methods include reverse abdominal breathing, cessation breathing and vocal breathing. Reverse abdominal breathing is used throughout the training of Liuzijue: on exhalation, the diaphragm rises, the abdominal muscles expand and the abdomen bulges. On inhalation, the diaphragm descends, the abdominal muscles contract and the abdomen retracts. Liuzijue training involves a conscious cessation of breathing, that is, allowing the breathing movement to stop between exhalation and inhalation, but this gap should not normally exceed 2 seconds and the participant should not be forced to hold his or her breath. Pronunciation breathing requires that the mouth shape, articulation and breathing are in harmony with each other. The focus of the Liuzijue training is on not only deep inhalation and slow exhalation, but also continuous, even and steady pronunciation. Liuzijue mainly focuses on breathing, supplemented by body movements, while other traditional qigong such as Taijiquan, Baduanjin and Wuqinxi pay less attention to breathing than the Liuzijue.

In COPD, weak and inefficient respiratory muscles can lead to breathing difficulties and movement difficulties. Respiratory muscle training counteracts the load by selectively exercising the respiratory muscles, thereby increasing strength, endurance and efficiency [18]. Liuzijue training combined with lip retraction help COPD patients to keep proper airway pressure and prevent airway closure, thereby improving gas exchange [19]. And its reverse abdominal breathing can effectively slow down expiratory flow to make breathing deep and slow. Thus changing the patient’s breathing pattern of high respiratory rate and low tidal volume [20–22]. Slow, deep breathing can also improve autonomic dysfunction in COPD patients [23, 24].

Liuzijue itself is a form of low-intensity aerobic exercise [25]. Aerobic exercise improves the function of respiratory and skeletal muscles, and enhances the patient’s exercise capacity [26]. Both low and high-intensity aerobic exercise can be clinically beneficial for COPD patients [27]. Besides, physical activity appeared to be an predictive indicator for COPD patients mortality [28]. Studies have shown that regular physical activity can reduce the hospitalization rate and mortality rate of COPD patients [29, 30]. As a form of exercise training, Liuzijue helps COPD patients establish a habit of exercise, which in turn improves patients’ outcomes.

The use of intention is also an integral part of Liuzijue training, which requires the trainer to relax the mind and body while imagining the essence of nature being inhaled while qi moves through the body and the turbid qi is exhaled. In the training of Liuzijue, the mind is focused on the breath, which is similar to the effects of mindful meditation [31]. Studies have shown that positive mindfulness training can influence the perceived internal awareness of key symptoms of COPD, such as breathlessness and coughing, resulting in better control of the patient’s symptoms [32] and relief of anxiety and depression in COPD patients [33].

This study has some innovative features. ACCP guidelines suggest that a longer pulmonary rehabilitation program (more than 12 weeks) will produce more sustained benefit than a shorter pulmonary rehabilitation program [27], so this study extends the intervention period to 3 months (12 weeks). Furthermore, this study extends observation period to 6 months (24weeeks) to observe whether patients benefited a long time after Liuzijue intervention. Maximal mid-expiratory flow (MMEF) is an indicator of the degree of small airway obstruction [34]. Previous studies have shown that COPD patients with a lower MMEF would exacerbated frequently [35]. This study attempts to observe whether Liuzijue training can improve the small airway function of patients. Previous studies have shown that labial contraction breathing has a more significant rehabilitation effect in COPD patients with low peak expiratory flow (PEF) [36]. What’s more, COPD patients have significantly improved their PEF after pulmonary rehabilitation with a thoracic exercise band [37]. This study observe whether Liuzijue training can improve patients’ PEF, or whether Liuzijue is more suitable for people with low PEF.

There are some limitations to this study. First, as the intervention factor is Liuzijue training, it is difficult to blind the participants, leading to biased results. Second, the compliance of patients with Liuzijue training at home will be lower than the outpatient or ward-based intervention. To deal with this, researchers will monitor patients’ WeChat applet training records, timely follow up patients with low compliance, and encourage them to overcome difficulties and complete daily training. Thirdly, Liuzijue is a traditional Chinese qigong, which may hinder its application and spreading in other countries.

## Supplementary Information

**Additional file 1. **SPIRIT 2013 checklist.

## Data Availability

The data used and/or analysed during the current study are available from the corresponding author on reasonable request.

## Abbreviations

COPD: chronic obstructive pulmonary disease
CG: control group
LG: Liuzijue group
FEV_1_%: percentage of forced expiratory volume in 1 second to predicted value
FEV_1_/FVC: ratio of forced expiratory volume in 1 second to forced vital capacity
MMEF: maximal mid-expiratory flow
PEF: peak expiratory flow
6MWT: 6-minute walk test
mMRC: modified British Medical Research Council
CAT: COPD assessment test score
AST: American Thoracic Society
ERS: European Respiratory Society
SGRQ: St. George’s respiratory questionnaire score.
